# Requirement of Peptidyl-Prolyl Cis/Trans isomerases and chaperones for cellular uptake of bacterial AB-type toxins

**DOI:** 10.3389/fcimb.2022.938015

**Published:** 2022-08-04

**Authors:** Katharina Ernst

**Affiliations:** Institute of Pharmacology and Toxicology, Ulm University Medical Center, Ulm, Germany

**Keywords:** bacterial AB-type toxin, chaperones, peptidyl-prolyl cis/trans isomerases, toxin inhibitors, pertussis toxin, cholera toxin, diphtheria toxin, clostridial binary toxins

## Abstract

Bacterial AB-type toxins are proteins released by the producing bacteria and are the causative agents for several severe diseases including cholera, whooping cough, diphtheria or enteric diseases. Their unique AB-type structure enables their uptake into mammalian cells *via* sophisticated mechanisms exploiting cellular uptake and transport pathways. The binding/translocation B-subunit facilitates binding of the toxin to a specific receptor on the cell surface. This is followed by receptor-mediated endocytosis. Then the enzymatically active A-subunit either escapes from endosomes in a pH-dependent manner or the toxin is further transported through the Golgi to the endoplasmic reticulum from where the A-subunit translocates into the cytosol. In the cytosol, the A-subunits enzymatically modify a specific substrate which leads to cellular reactions resulting in clinical symptoms that can be life-threatening. Both intracellular uptake routes require the A-subunit to unfold to either fit through a pore formed by the B-subunit into the endosomal membrane or to be recognized by the ER-associated degradation pathway. This led to the hypothesis that folding helper enzymes such as chaperones and peptidyl-prolyl cis/trans isomerases are required to assist the translocation of the A-subunit into the cytosol and/or facilitate their refolding into an enzymatically active conformation. This review article gives an overview about the role of heat shock proteins Hsp90 and Hsp70 as well as of peptidyl-prolyl cis/trans isomerases of the cyclophilin and FK506 binding protein families during uptake of bacterial AB-type toxins with a focus on clostridial binary toxins *Clostridium botulinum* C2 toxin, *Clostridium perfringens* iota toxin, *Clostridioides difficile* CDT toxin, as well as diphtheria toxin, pertussis toxin and cholera toxin.

## Introduction

Bacterial protein toxins cause several severe diseases, including botulism, cholera or whooping cough. These proteins are crucial virulence factors inducing characteristic cellular effects and thereby causing the typical toxin-associated clinical symptoms in humans or animals. A subgroup of bacterial protein toxins, the AB-type toxins, are secreted by the producing bacteria and therefore act independently from the bacteria. Moreover, AB-type toxins display a characteristic subunit structure and organization. They consist of two functional domains: an enzyme domain (A-domain) that exhibits enzymatic activity and a binding/translocation domain (B-domain) facilitating toxin binding to a cellular receptor as well as translocation of the enzyme domain into the cytosol. Therefore, these toxins act intracellular as enzymes and display high substrate specificity as well as outstanding potency.

**Figure 1:**
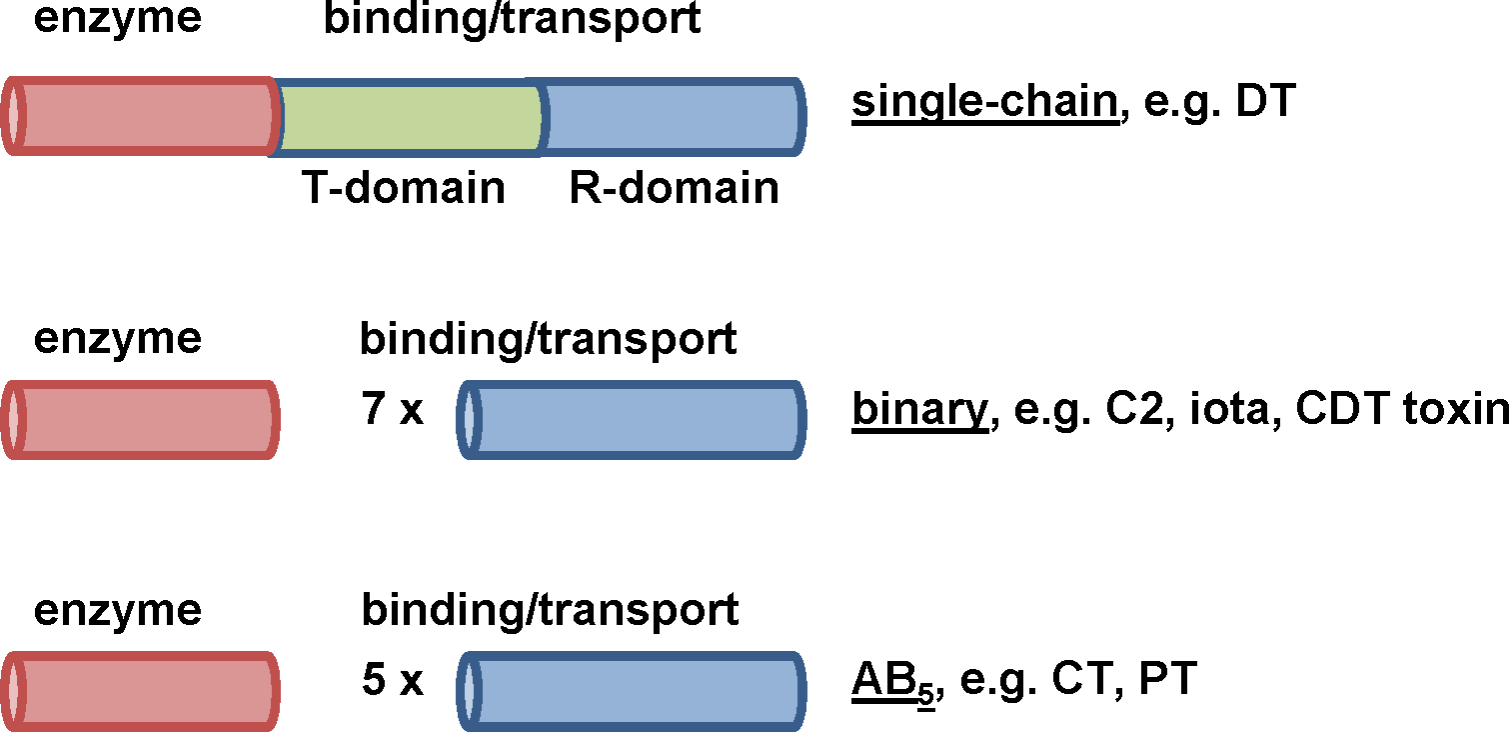
Different structures of bacterial AB-type toxins. Single-chain toxins consist of one single polypeptide chain and both domains, A-and B-domain, are located on this protein. For DT, the binding/transport B-domain can be subdivided in two further functional domains. The R-domain is responsible for receptor-binding and the T-domain is required for translocating the enzyme domain into the cytosol. Binary toxins express their functional domains on two separate proteins, then called A- and B-components. In solution or at the cell surface, seven B-components form a ring-shaped homooligomer to which the A- component binds. AB_5_-toxins such as CT or PT consist of one A-subunit and five B- subunits. The B-subunits form homo- (CT) or hetero- (PT) oligomers which non-covalently interact with the A-subunit. In contrast to binary toxins, formation of the AB_5_-holotoxin takes place in the periplasm of the producing bacteria, i.e. before the toxin is secreted.

Due to their structure, AB-type toxins are divided into single-chain, binary and AB_5_-type toxins (**Figure 1**). In single-chain toxins, such as the diphtheria toxin (DT), the A- and B-domain are located on one single protein (Collier, 1975). In binary toxins the two domains are located on two non-linked proteins called components. The *Clostridium (C.) botulinum* C2 toxin (Ohishi, 1983a; Ohishi, 1983b), the *C. perfringens* iota toxin (Stiles and Wilkins, 1986) and the *Clostridioides (C.) difficile* CDT toxin (Perelle et al., 1997) are constructed in a binary manner. Only if both, the A- and B-component, are present, the formed holotoxin elicits cytotoxicity on the target cell. AB_5_ toxins such as the *Bordetella (B.) pertussis* toxin (PT) or the *Vibrio (V.) cholerae* cholera toxin (CT) consist of one A-subunit and five B-subunits secreted by the bacteria as a non-covalently linked holotoxin (Hirst and Holmgren, 1987; Stein et al., 1994).

**Figure 2:**
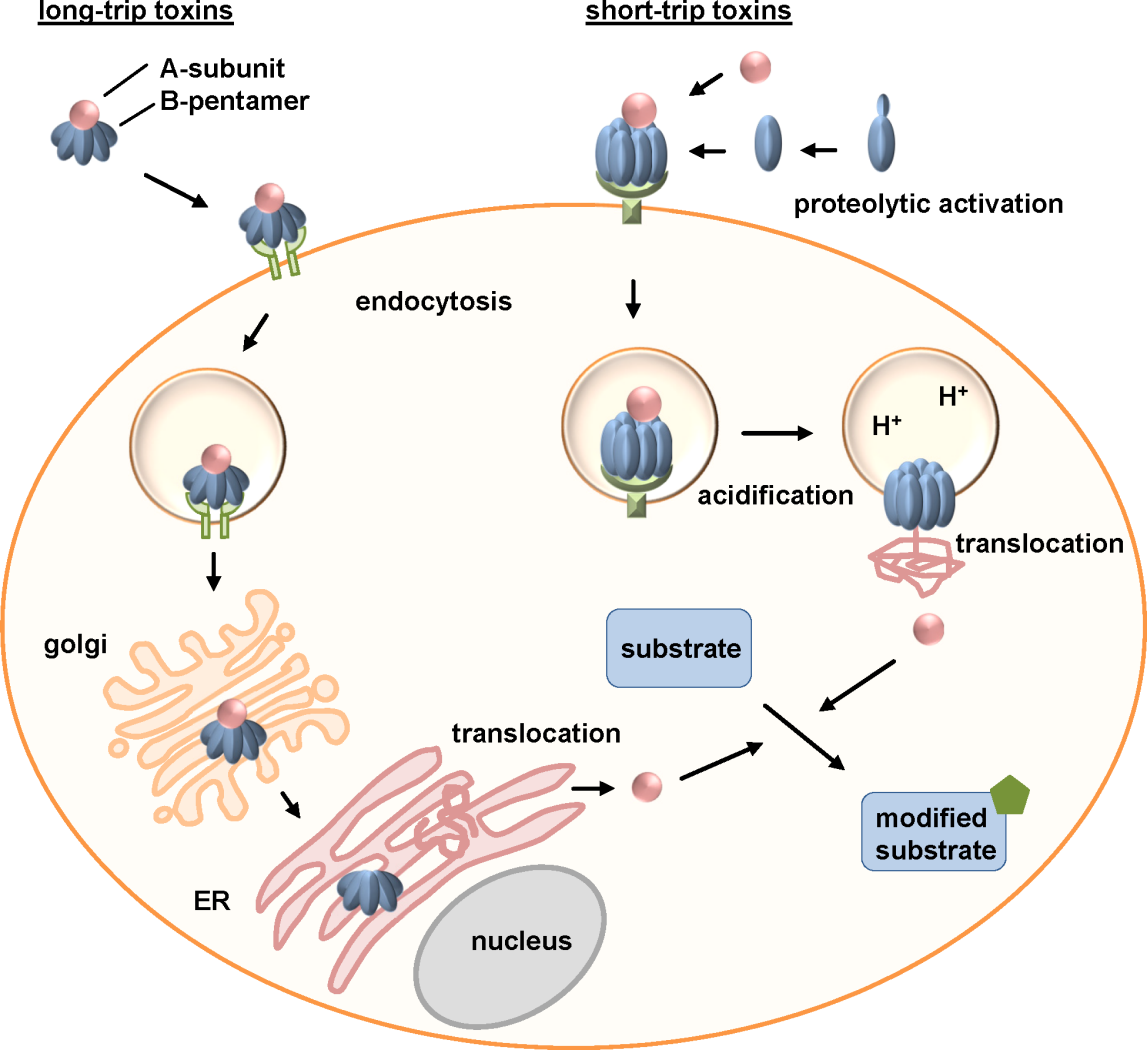
Uptake and intracellular routes of bacterial AB-type toxins. Long-trip toxins that for example comprise the AB_5_-toxins pertussis and cholera toxins (exemplarily depicted in this figure), bind to a cellular receptor *via* their B-subunits. After receptor-mediated endocytosis, long-trip toxins travel through the Golgi to the ER. Here, the enzyme subunit is released, unfolds and is transported *via* the ER-associated degradation (ERAD) pathway into the cytosol. Short-trip toxins, including amongst others clostridial binary toxins (exemplarily depicted in this figure) and diphtheria toxin, also bind to a receptor on the cell surface followed by receptor-mediated endocytosis. Acidification of endosomes leads to pore formation of the B-subunit due to conformational changes and unfolding of the A-subunit. The enzyme subunit translocates through the pores into the cytosol of cells. Here, long- and short-trip toxins enzymatically modify their specific substrates causing cellular effects and thereby clinical symptoms of the respective disease (Barth et al., 2004; Wernick et al., 2010; Barth and Ernst, 2016; Teter, 2019).

AB-type toxins have a sophisticated uptake mechanism to enter the target cell cytosol. Due to the different intracellular trafficking routes, AB-type toxins are distinguished in short- and long-trip toxins (**Figure 2**). Short-trip toxins, including DT or clostridial binary toxins, enter cells *via* receptor-mediated endocytosis. Acidification of these toxin-loaded endosomes leads to conformational changes of the toxin. The binding/translocation subunit inserts hydrophobic regions into the endosomal membrane, thereby facilitating the translocation of the enzyme domain into the cytosol. After receptor-mediated endocytosis, long-trip toxins, including PT and CT, are transported retrogradely from endosomes through the Golgi apparatus to the endoplasmic reticulum (ER). Their enzymes subunits are translocated from the ER to the cytosol.

AB-type toxins display different enzymatic activities such as protease (e.g. botulinum and tetanus neurotoxins) or glucosyltransferase (e.g. *Clostridioides difficile* TcdA and TcdB) activities. Many medically relevant toxins such as DT, PT, CT and binary clostridial toxins are ADP-ribosyltransferases which covalently transfer an ADP-ribose moiety from their co-substrate NAD^+^ to their specific substrates. The substrates of ADP-ribosyltransferases include amongst other the elongation factor 2, substrate of DT, α-subunits of inhibitory G proteins (Gαi), substrate of PT, α-subunits of stimulatory G proteins (Gαs), substrate of CT and G-actin, substrate of clostridial binary toxins. Modification of these substrates leads to specific cellular effects resulting in the respective clinical symptoms.

During the last years, it was shown that several AB-type toxins rely on the activity of host cell peptidyl-prolyl cis/trans isomerases (PPIases) as well as chaperones of the heat shock protein family for their cellular uptake in particular to facilitate the translocation of their enzyme subunits from cellular compartments such as endosomes into the cytosol (Ernst et al., 2017b). In this review the requirement and role of PPIases and chaperones for the cellular uptake of AB-type toxins are described.

## Host cell PPIases and chaperones

**Figure 3:**
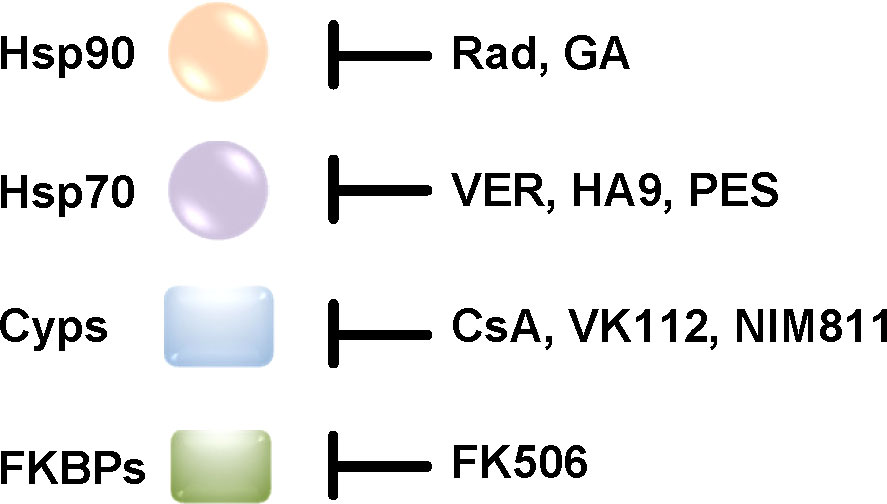
The activity of chaperones and PPIases is inhibited by specific pharmacological inhibitors in cells. Rad/GA and VER block the ATPase activity of Hsp90 and Hsp70, respectively. HA9 inhibits the substrate binding domain of Hsp70. PES inhibits Hsp70 chaperone activity. CsA and FK506 inhibit the PPIase activity of Cyps and FKBPs, respectively. CsA and FK506 are licensed drugs with immunosuppressive effects. VK112 and NIM811 are derivatives of CsA that were specifically designed to still inhibit the PPIase activity without immunosuppression which would be desirable for a potential therapeutic option of Cyp-inhibition in the context of an infection with toxin-producing bacteria (Rosenwirth et al., 1994; Williamson et al., 2009; Prell et al., 2013; Schlecht et al., 2013; Ernst et al., 2016; Biebl and Buchner, 2019; Hähle et al., 2019; Braun et al., 2022). (Rad, radicicol; GA, geldanamycin; VER, VER-155008; PES, 2-phenylethynesulfonamide; CsA, cyclosporine A; Cyps, cyclophilins; FKBPs, FK506 binding protein).

Molecular chaperones are essential to eukaryotic cells because they enable protein homeostasis, which means maintaining the integrity of the cellular protein network. In this context the ability to adapt to changing environmental conditions is of great importance. To this end, chaperones not only interact and facilitate the correct folding of newly synthesized proteins but also help to avoid aggregation of proteins especially under stress conditions such as increases in temperature. This review focuses on two protein families that are involved in protein folding: heat shock proteins (Hsps) and peptidyl-prolyl cis/trans isomerases (PPIases). Notably, an interplay of Hsps and PPIases has been shown, for example for the activation and folding of steroid hormone receptors (Pratt and Toft, 1997). The role of the heat shock proteins Hsp90 and Hsp70 as well as of PPIases of the cyclophilin (Cyp) and FK506 binding protein (FKBP) families for the uptake of several bacterial AB-type toxins was investigated during the last years (Ernst et al., 2017b). Therefore, the use of specific pharmacological inhibitors of these chaperones and PPIases was crucial to determine their requirement for toxin uptake (**Figure 3**).

### Hsp90 and Hsp70

Molecular chaperones act as buffers against changing environmental conditions such as temperature increases which originates the name heat shock proteins (Ritossa, 1996). However, Hsp90 is not only expressed under heat shock conditions but is one of the most abundant proteins in the cytosol under physiological conditions (Schopf et al., 2017). As a highly conserved protein, it is involved in various processes in the cell such as stress response, cell cycle control and survival, as well as hormone signaling. Confirming the correct conformation and activation of proteins is a key function of Hsp90. Hundreds of Hsp90 substrate proteins, also called clients, are known (Biebl and Buchner, 2019). Hsp90 contains three functional domains. The C-terminal dimerization site is responsible for formation of Hsp90 homodimers (Pearl and Prodromou, 2000). The middle domain connects to the N-terminal nucleotide binding domain which binds ATP resulting in a closed conformation of the Hsp90 dimer. ATP hydrolysis by an intrinsic ATPase activity facilitates conformational changes of the client protein and release of the folded client (Biebl and Buchner, 2019). Hsp90 often interacts with various co-chaperones that influence the processed clients as well as regulate the ATPase activity. An MEEVD-motif at the dimerization domain allows the interaction of Hsp90 with co-chaperones that contain tetratricopeptide repeat (TPR) domains. These co-chaperones include Hsp70 Cyp40, FKBP51 or FKBP52 (Ratajczak and Carrello, 1996). Thereby, an Hsp90 machinery is formed which facilitates the folding and activation in a stepwise cycle that is best investigated for the steroid hormone receptors as client proteins (Pratt and Toft, 2003; Biebl and Buchner, 2019). Since Hsp90 is involved in diseases such as cancer, viral infections or neurodegenerative diseases, pharmacological targeting of Hsp90 activity was investigated in the last years. Inhibitors such as radicicol (Rad) or geldanamycin (GA) bind selectively to the Hsp90 ATP-binding pocket and thereby inhibit Hsp90 activity (Pratt and Toft, 2003; Biebl and Buchner, 2019). Although derivates of Rad and GA tested in phase I and II clinical trials were discontinued due to lack of efficacy at the tolerated doses, these inhibitors are valuable tools to discover and unravel the role of Hsp90 in cellular processes such as uptake of bacterial AB-type toxins (Ernst et al., 2017b; Biebl and Buchner, 2019).

Another prominent member of the molecular chaperone family and a co-chaperone of Hsp90 is Hsp70, an abundant, highly conserved protein that is involved in a wide range of cellular activities such as folding of newly synthesized proteins, translocation of polypeptide chains into mitochondria or the ER or prevention of aggregation and refolding of misfolded proteins (Rosenzweig et al., 2019). Assistance of protein translocation mostly occurs due to entropic pulling by Hsp70, which means that Hsp70 binds the translocating protein very close to the translocation pore. Thereby, the Brownian movement is limited, and a one-way pulling motion is generated (Clerico et al., 2015; Finka et al., 2015; Rosenzweig et al., 2019). A comparable mechanism might be involved in Hsp70-assisted translocation of bacterial AB-type toxins. Hsp70 encompasses four domains: The N-terminal nucleotide binding domain, the substrate binding domain, a helical lid domain and a disordered C-terminal tail (Rosenzweig et al., 2019). ATP binding and hydrolysis is crucial for Hsp70 function and is prevented by the inhibitor VER-155008 (VER) (Williamson et al., 2009). The substrate binding site of Hsp70 is inhibited by the 9-aminoacridizinium derivative HA9, which also showed a higher specificity for Hsp70 compared to its constitutive from Hsc70 (Ernst et al., 2016; Ernst et al., 2017a). Another inhibitor 2-phenylethynesulfonamide (PES) was shown to inhibit Hsp70 chaperone activity without affecting its substrate binding site (Schlecht et al., 2013). These pharmacological inhibitors were crucial to investigate the involvement of Hsp70 during uptake of bacterial AB-type toxins such as DT, PT or CT.

### Cyclophilins and FKBPs

Cyps and FKBPs are PPIases that catalyze the cis/trans isomerization of prolyl-bonds. This often represents a rate-limiting step in protein folding. The highly conserved families of Cyps and FKBPs comprise several isoforms with single- or multidomain structures (Schiene-Fischer, 2015; Hähle et al., 2019; Braun et al., 2022). The single-domain isoform FKBP12 as well as CypA contain a single PPIase domain and are involved in various cellular processes such as signal transduction or oxidative stress response (Schiene-Fischer, 2015). Cyp40 as well as FKBP51 and FKBP52 represent multidomain isoforms that contain three tetratricopeptide repeat (TPR) domains in addition to the PPIase domain (Schiene-Fischer, 2015; Hähle et al., 2019). Thereby, interactions with proteins containing an MEEVD motif such as Hsp90 or Hsc70 are facilitated (Davis et al., 2010; Schiene-Fischer, 2015). Binding of Cyps or FKBPs to the Hsp90 machinery affects Hsp90 activity and was shown for the activation and folding of steroid hormone receptors (Prodromou et al., 1999). The activity of Cyps and FKBPs is inhibited by cyclosporine A (CsA) and FK506, respectively. CsA and FK506 both mediate an immunosuppressive effect because the complex of CsA-Cyps as well as FK506-FKBPs binds to the protein phosphatase calcineurin. Thereby, calcineurin is inhibited and no longer able to dephosphorylate the transcription factor nuclear factor of activated T-cell (NF-AT). NF-AT then remains in the cytosol unable to promote the transcription of interleukins which results in decreased T-lymphocyte activation (Liu et al., 1991; Braun et al., 2022). FK506, also known as tacrolimus, and CsA are licensed immunosuppressive drugs that are applied for example after organ transplantation to prevent organ rejection. Comparable to Hsp90 and Hsp70 inhibitors, CsA and FK506 were used to investigate the role of Cyps and FKBPs during uptake of bacterial AB-type toxins. Since CsA and FK506 are both licensed drugs, novel derivatives that were specifically modified to lack the immunosuppressive effect (see also 3.5) are of particular interest as a potential basis for novel therapeutic strategies against disease-causing toxins that depend on Cyps and FKBPs for their cellular uptake.

## Role of PPIases and chaperones for the uptake of bacterial AB-type toxins

To achieve their cytotoxic effects and thus to cause clinical symptoms, AB-type employ a very elaborate uptake mechanism that involves the step of membrane translocation either from endosomes or other intracellular compartments such as the ER. Therefore, the enzyme subunits of these toxins have to be unfolded to fit through narrow translocation pores either formed by the B-subunits of the toxin itself or by using translocation pores of the ER. In the cytosol, the enzyme subunits have to be refolded to achieve their native and active conformation. Thus, the hypothesis was established that host cell protein folding helper enzymes such as PPIases and heat shock proteins are required to facilitate the directed translocation of the enzyme subunits to the cytosol and to assist their refolding into an active conformation. In the following, the role of PPIases and heat shock proteins during cellular uptake of clostridial binary toxins (see 3.1), DT (see 3.2), PT (see 3.3) and CT (see 3.4) is described in more detail.

### Clostridial binary toxins

**Figure 4:**
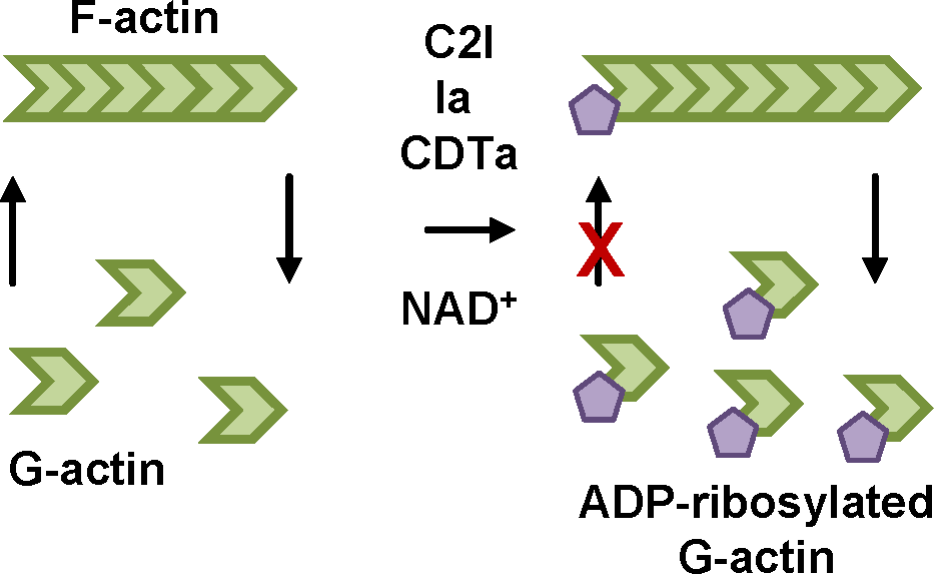
ADP-ribosylation of G-actin by clostridial binary leads to depolymerization of F-actin. The enzyme components of C2, iota and CDT toxin, C2I, Ia and CDTa, respectively, covalently transfer an ADP-ribose moiety from their co-substrate NAD^+^ onto their specific substrate monomeric actin (G-actin). If ADP-ribosylated G-actin binds to an actin filament (F-actin) no additional G-actin molecule can be attached because of steric hinderance. Due to the dynamic structure of F-actin, the other end of the filament continues to depolymerize thereby making more G-actin molecules available for ADP-ribosylation by the toxin enzymes. Therefore, in the end the complete actin cytoskeleton is depolymerized and most of the G-actin is ADP-ribosylated. In adherent cells, this leads to a characteristic cell rounding that is used as a specific and highly sensitive readout for intoxication in cells (Wegner and Aktories, 1988).

Clostridial binary toxins are short-trip toxins that translocate their enzyme components from acidified endosomes into the cytosol (**Figures 1**, **2**). In the cytosol, the A-components enzymatically modify G-actin resulting in the depolymerization of the actin cytoskeleton (**Figure 4**) (Barth et al., 2004). The *C. botulinum* C2 toxin that represents the prototype of this toxin family causes necrosis and hemorrhagic lesions in the intestinal mucosa in animal experiments with mice (Ohishi et al., 1980; Simpson, 1982; Ohishi, 1983a; Ohishi, 1983b). C2-induced fluid accumulation in the intestinal loop of pheasants and chickens was also observed (Kurazono et al., 1987). For *C. perfringens* iota toxin, enterotoxicity in livestock such as lambs and calves was reported (Songer, 1996; Billington et al., 1998). Iota toxin and *C. difficile* CDT toxin share a high extent of sequence homology and their A- and B-components are interchangeable (Perelle et al., 1997; Barth et al., 2004). *C. difficile* is clinically highly relevant and causes antibiotic-associated diarrhea as well as pseudomembranous colitis (Kampouri et al., 2021). Increasing case numbers have been observed during the last year, especially in North America and Europe. In most cases, *C. difficile* infections are healthcare associated and can lead to severe complications that are associated with high mortality. Infections are treated with specific antibiotics such as metronidazole, vancomycin or fidaxomicin. However, 10-30 % of patients develop at least one recurrence of disease and the risk for recurrence increases with each episode (Leffler and Lamont, 2015; Kampouri et al., 2021). The main virulence factors of *C. difficile* are the AB-type toxins TcdA and TcdB that glucosylate Rho-GTPases thereby disturbing Rho-dependent signaling pathways and resulting in destruction of the actin cytoskeleton (Aktories et al., 2017). Treatment with the monoclonal antibody bezlotoxumab directed against TcdB in addition to antibiotic therapy has been shown to reduce the risk of recurrence of *C. difficile* infection. Significant benefits were especially observed in high risk patients (e.g. age >65) (Johnson and Gerding, 2019). Hypervirulent *C. difficile* strains that are associated with more antibiotic resistances, increased production of TcdA and TcdB and overall increased disease severity and mortality additionally produce the CDT toxin (Aktories et al., 2018). Although the precise role of CDT as a virulence factor is still under investigation, it was shown that in addition to destruction of the actin cytoskeleton, CDT induces long microtubule-based protrusions in target cells. In a mice infection model, *C. difficile* bacteria get caught in these protrusions thereby increasing colonization of the bacteria in the gut (Schwan et al., 2009; Schwan and Aktories, 2017). Moreover, CDT was demonstrated to suppress protective colonic eosinophilia (Cowardin et al., 2016).

Structures and cellular uptake mechanisms of C2, iota and CDT toxins are widely comparable. CDT consists of the binding/translocation component CDTb and the enzyme component CDTa. After proteolytic activation, seven CDTb monomers assemble to form a ring-shaped CDTb heptamer, also called pre-pore (Anderson et al., 2020). The CDTb heptamer binds to a specific receptor, the lipolysis-stimulated lipoprotein receptor (LSR), on the cell surface (Papatheodorou et al., 2011). The LSR is also the specific receptor of the iota toxin (Nagahama et al., 2018). Additionally, CD44 was identified as a co-receptor for CDT (Wigelsworth et al., 2012). C2 toxin binds to carbohydrate structures on the cell surface (Eckhardt et al., 2000). CDTa then binds to the CDTb heptamer and after receptor-mediated endocytosis, acidification of endosomes triggers translocation of CDTa through a pore formed by the CDTb heptamer into the cytosol (Sheedlo et al., 2020). A prerequisite for translocation is the unfolding of CDTa to fit through the narrow CDTb-pore. For C2 toxin, the inner diameter of the heptamer pore was shown to be 2-3 nm (Schleberger et al., 2006). In the cytosol, the clostridial enzyme components covalently transfer an ADP-ribose moiety from their co-substrate NAD^+^ onto their specific substrate monomeric actin (G-actin) (**Figure 4**) (Aktories et al., 1986; Popoff et al., 1988; Schering et al., 1988; Gülke et al., 2001). Interaction of ADP-ribosylated G-actin with the growing end of filamentous actin (F-actin) is reduced due to steric hinderance (Wegner and Aktories, 1988). At the other end of the actin filament, release of G-actin is still ongoing. In the end, this results in complete depolymerization of the actin cytoskeleton and leads to rounding of cultured, adherent cells.

The first host cell chaperone that was identified to play a role in toxin uptake was Hsp90. In 2003 it was shown for a diphtheria fusion toxin as well as for the C2 toxin that Hsp90 was required for the uptake of the toxin enzyme components into the cytosol (Haug et al., 2003b; Ratts et al., 2003). Later, the requirement of Hsp90 was also shown for iota and CDT toxin (Haug et al., 2004; Kaiser et al., 2011). The activity of Hsp90 in cells was inhibited by the specific pharmacological inhibitors Rad and GA. Hsp90 inhibition significantly delayed the intoxication of cells with C2, iota and CDT toxins (Haug et al., 2003a; Haug et al., 2004; Kaiser et al., 2011). This was first shown by analyzing the toxin-induced specific changes in cell morphology. Due to ADP-ribosylation of G-actin, the cytoskeleton of cells is destroyed. This leads to a characteristic cell rounding that can be observed in adherent cells such as Vero, HeLa or CaCo-2 cells. Cells pre-incubated with Hsp90 inhibitors prior to intoxication revealed less rounded cells compared to samples treated with toxin only (Haug et al., 2003a; Haug et al., 2004; Kaiser et al., 2011). Hsp90 inhibitors also led to a reduced amount of ADP-ribosylated G-actin in toxin-treated cells indicating inhibition of intoxication. To unravel which step of toxin uptake and mode of action requires the activity of Hsp90, a set of experiments was performed with each experiment addressing single steps of the uptake or mode of action. Thereby, it was shown that upon Hsp90 inhibition less enzyme molecules reach the cytosol and that specifically the translocation of the enzyme component from endosomes to the cytosol is facilitated by Hsp90. Other steps such as receptor-binding, endocytosis, or *in vitro* enzyme activity were not affected by Hsp90 inhibitors (Haug et al., 2003a; Haug et al., 2004; Kaiser et al., 2011).

Hsp90 is known to interact with other protein folding helper enzymes in a concerted manner to facilitate protein folding. Interaction of Hsp90 with for example Hsp70, Cyps and FKBPs is mediated by the MEEVD motif in Hsp90 and the TPR regions in the binding partners (Pratt and Toft, 1997; Biebl and Buchner, 2019). The reported concerted action of an Hsp90-multichaperone complex led to the hypothesis that the uptake of bacterial proteins might also rely on further co-chaperones in addition to Hsp90. Accordingly, the requirement of Cyps and FKBPs for translocation of C2 toxin and then for iota and CDT toxin was shown (Kaiser et al., 2009; Kaiser et al., 2011; Kaiser et al., 2012; Ernst et al., 2015). The activity of Cyps and FKBPs in cells was inhibited by CsA and FK506, respectively. Inhibition of Cyps and FKBPs also resulted in reduced amount of enzyme components in the cytosol due to impairment of translocation from endosomes. Like Rad, CsA and FK506 did not inhibit receptor-binding, endocytosis, or enzyme activity *in vitro* (Kaiser et al., 2009; Kaiser et al., 2011; Kaiser et al., 2012; Ernst et al., 2015). Moreover, the protein-protein interaction of the enzyme components with chaperones and PPIases was investigated. This revealed a direct interaction of the enzyme components of C2, iota and CDT toxin with Hsp90 and different isoforms of Cyps and FKBPs. Co-precipitation, dot blot analysis as well as isothermal titration calorimetry revealed the small isoform CypA as well as the multi-domain isoform Cyp40 as interaction partners. A dissociation constant of 1 µM was determined for the interaction of C2I with Cyp40 (Ernst et al., 2015). For the family of FKBPs, only multi-domain isoform FKBP51 but not the small isoform FKBP12 showed binding to the enzyme components of clostridial binary toxins (Kaiser et al., 2012).

Another co-chaperone of Hsp90, Hsp70, was also shown to be required for the translocation process of C2, iota and CDT toxin enzyme components (Ernst et al., 2016; Ernst et al., 2017a). By using different inhibitors of Hsp70, it was shown that both the substrate binding domain, inhibited by the novel inhibitor HA9, and the ATP-binding domain, inhibited by VER, are required for uptake of C2, iota and CDT toxin. A direct interaction of the enzyme components with Hsp70 as well as with the constitutive form Hsc70 was demonstrated (Ernst et al., 2016; Ernst et al., 2017a). Interestingly, the interaction of the enzyme components with Hsp70, Hsc70 and also with FKBP51 and Cyp40 was enhanced when the enzyme components were treated with guanidinium hydrochloride to induce their denaturation (Kaiser et al., 2012; Ernst et al., 2015; Ernst et al., 2016; Ernst et al., 2017a). During cellular uptake of the toxins, the only time the enzyme components are in an unfolded conformation is during the translocation from endosomes to the cytosol. Therefore, enhanced interaction of the denatured i.e. unfolded enzymes with chaperones and PPIases underlines their requirement to facilitate this step of toxin uptake. So far, an interaction was shown by *in vitro* protein-protein interaction analysis such as dot blot or isothermal titration calorimetry. With the fluorescence-based proximity ligation assay, it was possible to show that the enzyme component C2I of C2 toxin is in close proximity (max. 40 nm distance) with Hsp90, Hsp70, Cyp40 and FKBP51 in cells (Ernst et al., 2017a; Ernst et al., 2018b). This assay is based on immunofluorescence with an internal signal amplification mechanism to overcome the detection limit in fluorescence microscopy to visualize single molecule interactions.

The importance of Hsp70 activity for the uptake of the *C. difficile* CDT toxin was additionally demonstrated in a complex human intestinal organoid model, so called miniguts (Ernst et al., 2017a). Miniguts are derived from hair sheet keratinocyte cultures of healthy donors. Cellular reprogramming results in induced pluripotent stem cells that were differentiated into intestinal organoids. Miniguts recapitulate basic characteristics of the human gut such as crypt-like structures and a polarized intestinal epithelium with epithelial as well as non-epithelial cell types e.g. goblet cells. When treated with CDT toxin, miniguts lose their F-actin structure due to ADP-ribosylation of G-actin (Ernst et al., 2017a). Consequently, the integrity of miniguts is severely compromised shown also by disorganization of the adhesion protein E-cadherin. In the presence of the Hsp70 inhibitor VER, F-actin, E-cadherin and the overall structure of miniguts was more preserved and resembled more closely to the structure of untreated control miniguts (Ernst et al., 2017a).

**Figure 5:**
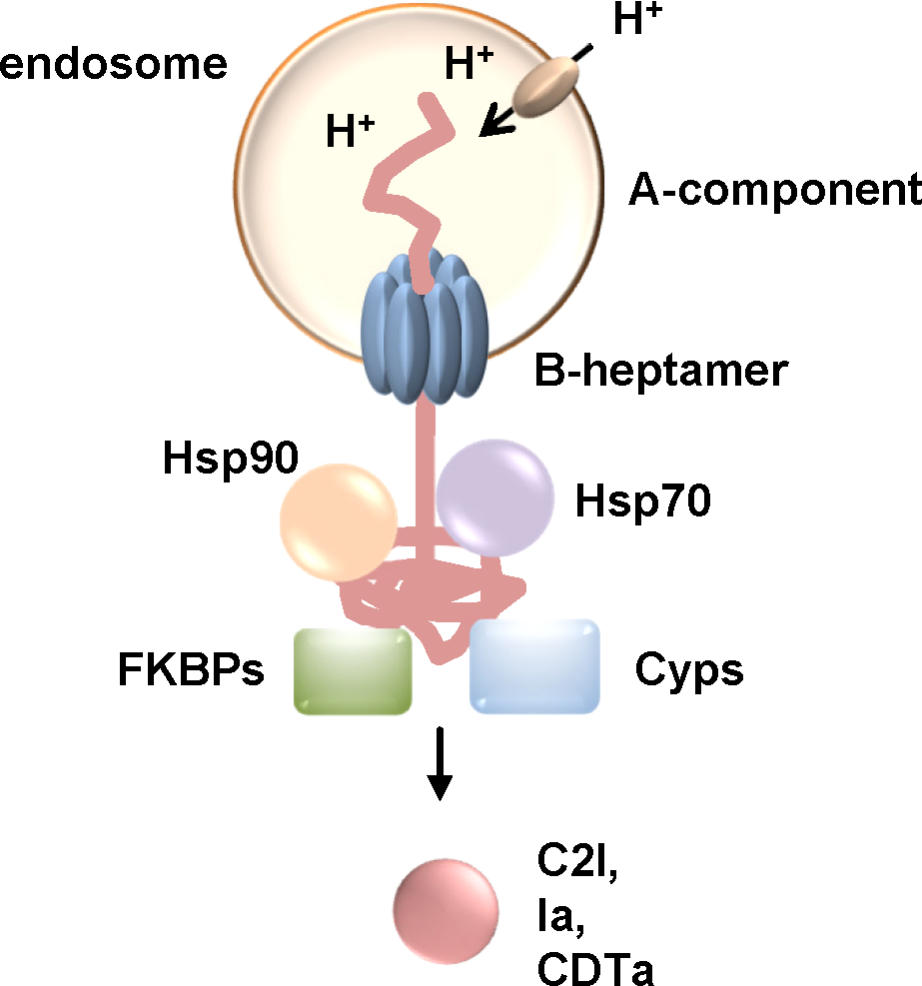
The activity of Hsp90, Hsp70, Cyps and FKBPs is required for the translocation of the enzyme components C2I, Ia and CDTa of clostridial binary C2, Iota and CDT toxins, respectively. These clostridial binary toxins are taken up *via* receptor-mediated endocytosis. Acidification of endosomes leads to conformational changes resulting in pore formation of the B-heptamer into the endosomal membrane. The enzyme components are at least partially unfolded and translocate through the narrow pore into the cytosol. If the activity of Hsp90, Hsp70, Cyps or FKBPs is inhibited by specific pharmacological inhibitors, less enzyme molecules reach the cytosol and cells are protected from intoxication (Haug et al., 2003a; Haug et al., 2004; Kaiser et al., 2009; Kaiser et al., 2011; Kaiser et al., 2012; Ernst et al., 2015; Ernst et al., 2016; Ernst et al., 2017a). (Cyps, cyclophilins; FKBPs, FK506-binding proteins).

Since the requirement of Hsp90, Hsp70, Cyps and FKBPs for uptake of clostridial binary toxins was shown (**Figure 5**), the effect of simultaneous inhibitions of these host cell factors was investigated. Enhanced inhibition of intoxication of cells with C2, iota or CDT toxin was observed if CsA and FK506 or CsA and Rad were combined compared to application of the single inhibitors (Kaiser et al., 2009; Kaiser et al., 2011; Kaiser et al., 2012). Combination of all four inhibitors, Rad, VER, CsA and FK506, resulted in an even stronger inhibition compared to the single substances as well as to the combination of two or even three inhibitors (Ernst et al., 2018b; Ernst et al., 2021b). Moreover, by combining the inhibitors it was possible to reduce the concentration of each inhibitor and still achieve a protective effect against intoxication (Ernst et al., 2021b).

Taken together, it was shown that the clostridial binary toxins C2, iota and CDT toxin require Hsp90, Hsp70, Cyps and FKBPs for translocation of their enzyme components from endosomes to the cytosol. Translocation through the pores might be due to Brownian motion of the unfolded enzyme. Binding of Hsps and PPIases to the enzyme components probably causes a directed movement into the cytosol by preventing the enzyme from sliding back into the endosomes due to Brownian back-and-forth motion. Inhibition by specific pharmacological inhibitors of Hsps and PPIases therefore reduced the amount of enzyme components in the cytosol of target cells and delayed intoxication. Moreover, Hsp70 inhibition also protected human miniguts from CDT-intoxication. The current therapeutic strategy against *C. difficile* associated diseases such as severe pseudomembranous colitis comprises antibiotic treatment as well as a neutralizing antibody against TcdB, one of the main virulence factors. However, antibiotic resistance of *C. difficile* is an increasing problem and infection with *C. difficile* reoccurs in ca. 30% of patients (Kampouri et al., 2021). Therefore, additional strategies targeting further toxins of *C. difficile* such as CDT toxin might be beneficial for sustained curative treatment. Inhibition of Hsps or PPIases to prevent cellular uptake of toxins and therefore cellular effects might provide a basis for development of novel therapeutic strategies to treat toxin-mediated diseases.

### Diphtheria toxin

DT is produced by *Corynebacterium diphtheriae* colonizing the upper respiratory tract and is the causative agent for the disease diphtheria (Pappenheimer, 1977). DT is one of the best characterized bacterial toxins and was first described in 1888 (Roux and Yersin, 1888). Fever, swollen glands, and sore throat are symptoms of diphtheria. Moreover, formation of an adherent membrane, the so-called pseudomembrane, impairs breathing as well as swallowing. If the inflammation also affects the nasal cavity and larynx, the obstruction of the airways can get more severe resulting in dyspnea, suffocation and death. In severe cases, DT is also distributed *via* the bloodstream which then can cause myocarditis or neuropathy (Atkinson et al., 2007; Ott et al., 2022). Before the introduction of a vaccination against diphtheria in the 1920s, children were mainly affected by the disease with a high prevalence as well as mortality (English, 1985; Atkinson et al., 2007; Ott et al., 2022). Despite the availability of a vaccination, diphtheria is not eradicated and outbreaks still occurred recently, for example in Bangladesh, Haiti or South Africa (Clarke et al., 2019; Ott et al., 2022). Moreover, case numbers worldwide have been increasing during the last years. Thus, *C. diphtheriae* is considered to be a re-emerging pathogen (Ott et al., 2022). The current therapy consists of antibiotics in combination with neutralizing antibodies against circulating DT. Strategies to neutralize or inhibit the already endocytosed DT are not available.

DT is a single chain AB-type toxin containing the enzymatically active A-domain, DTA as well as the transport domain DTB (**Figure 1**) (Collier and Kandel, 1971). DTB itself comprises two functional domains. The receptor-binding domain, R-domain, is responsible for binding to a specific receptor, the heparin-binding epidermal growth factor-like growth factor (HB-EGF) (Choe et al., 1992; Naglich et al., 1992). The transmembrane T-domain of DTB is important for the translocation of DTA into the cytosol. Recently, it was shown that the T-domain also partly facilitates binding of DT to cells (Fellermann et al., 2020). A loop of 14 amino acids as well as a disulfide bond connect DTA and DTB (Gill and Pappenheimer, 1971). As a short-trip toxin, DT is taken up by receptor-mediated endocytosis and translocates its enzyme domain from early acidified endosomes into the cytosol (**Figure 2**). Furin proteases cleave DTA from DTB. However, in endosomes, the domains are still connected by the disulfide bond (Gill and Pappenheimer, 1971). Low endosomal pH causes unfolding of the T-domain and its insertion into the endosomal membrane thereby forming a membrane pore (Boquet et al., 1976; Donovan et al., 1981; Kagan et al., 1981). DTA translocates through the pore into the cytosol (Papini et al., 1993b; Papini et al., 1993a; Lemichez et al., 1997) which requires at least partial unfolding of DTA (Falnes et al., 1994; Falnes and Olsnes, 1995). DTA is finally released after the reduction of the interchain disulfide bond during or after translocation (Moskaug et al., 1987; Papini et al., 1993b; Madshus, 1994; Schnell et al., 2015). In the cytosol, DTA ADP-ribosylates the cytosolic elongation factor 2 (EF-2) (Collier and Cole, 1969; Pappenheimer, 1977). EF-2 is required for protein synthesis in eukaryotic cells and ADP-ribosylation leads to its inactivation. This causes an arrest in chain elongation during protein translation, leading in the end to cell death by apoptosis (Collier and Cole, 1969).

A functional role of Hsp90 for translocation of DTA into the cytosol was first demonstrated in 2003 (Ratts et al., 2003). Therefore, a fusion protein, DAB_389_IL-2, was used where the receptor-binding domain was substituted for human interleukin-2 (IL-2) to specifically target cells expressing IL-2 receptors (Ratts et al., 2003). A cytosolic translocation factor complex was shown to be required for DTA translocation. By applying mass spectrometry sequencing, Hsp90 as well as thioredoxin reductase were identified from these protein complexes. This protein complex containing Hsp90 was required for translocation of DTA from partially purified endosomal vesicles that were pre-loaded with DT. Further studies were conducted with another fusion protein consisting of the enzyme domain DTA fused to the N-terminal part of the lethal factor which is the enzyme component of the binary anthrax toxin (Dmochewitz et al., 2011). The N-terminus of the lethal factor called LF_N_ has no enzyme activity but still interacts with its B-component, the protective antigen (PA). Thereby, the resulting fusion protein, termed LF_N_DTA, can be transported into cells *via* PA and then ADP-ribosylates the DT-substrate EF-2. A direct interaction of LF_N_DTA or DTA with Hsp90 and CypA was shown. Moreover, CsA and Rad inhibited intoxication of cells with PA + LF_N_DTA as well as translocation of LF_N_DTA into the cytosol demonstrating that Cyps and Hsp90 also facilitate the translocation of LF_N_DTA (Dmochewitz et al., 2011).

Later it was shown that Hsp70, Cyps and FKBPs facilitate the translocation of DTA into the cytosol besides Hsp90 (Schuster et al., 2017). Again, this was demonstrated by using specific pharmacological inhibitors of chaperone and PPIase activities which reduced morphological effects of intoxication, the amount of ADP-ribosylated EF-2 as well as the amount of DTA in the cytosol (Schuster et al., 2017). Enzyme activity of DTA *in vitro* as well as binding of DT to cells was not affected by chaperone/PPIases inhibitors. Direct evidence that chaperones/PPIases facilitate the pH-dependent translocation of DTA was given by mimicking the acidic endosomal conditions directly at the cytoplasmic membrane. Therefore, binding of DT is allowed at 4°C followed by a pulse with warm acidic medium. This leads to translocation of DTA directly across the cytoplasmic membrane, therefore bypassing other steps of intracellular trafficking. Inhibition of Hsp90, Hsp70, Cyps or FKBPs prevented translocation of DTA across the cytoplasmic membrane (Schuster et al., 2017). A direct interaction of DTA with different isoforms of chaperones and PPIases was shown by dot blot analysis and co-precipitation. Here too, the interaction was increased if DTA was denatured prior to dot blot interaction analysis. Moreover, DTA interacted with fragments of FKBPs that only contain the PPIase domain. However, this interaction was weaker compared to the full-length FKBPs indicating that other domains of the large FKBP isoforms, FKBP51 and 52, are required for interaction with DTA (Schuster et al., 2017).

Taken together, it was shown that DT requires the same set of Hsps and PPIases as the clostridial binary toxins for translocation of its enzyme domain into the cytosol. Inhibition of Hsp90, Hsp70, Cyps or FKBPs resulted in a reduced amount of DTA in the cytosol of target cells and delayed the intoxication of cells with DT (Ratts et al., 2003; Dmochewitz et al., 2011; Schuster et al., 2017)

### Pertussis toxin

PT is a major virulence factor of *B. pertussis* and is secreted during infection of the upper respiratory tract. The disease is highly transmissible and characterized by severe paroxysmal coughing typically lasting for several weeks. Complications such as vomiting or pneumothorax or in more severe cases pneumonia, seizures or apnea can occur. These complications can be life-threatening especially in newborns and infants (Mattoo and Cherry, 2005). A hallmark of severe infant disease is leukocytosis, the rapid and unregulated increase in circulating leukocytes. Leukocytosis is associated with PT expression as well as with poor disease outcome and death. In 2014, the number of pertussis cases in children <5 years of age was estimated at >24.1 million with 160,700 deaths worldwide by the WHO (Yeung et al., 2017). Despite high vaccination rates, especially in Western countries, case numbers increased during the last years, reaching an all-time high since vaccination has been introduced in the 1950s (Locht and Antoine, 2021). Therapeutic options against pertussis symptoms are limited. Antibiotic therapy is important to prevent further transmission but in most cases, is started too late to alleviate patients from symptoms (Mattoo and Cherry, 2005).

**Figure 6:**
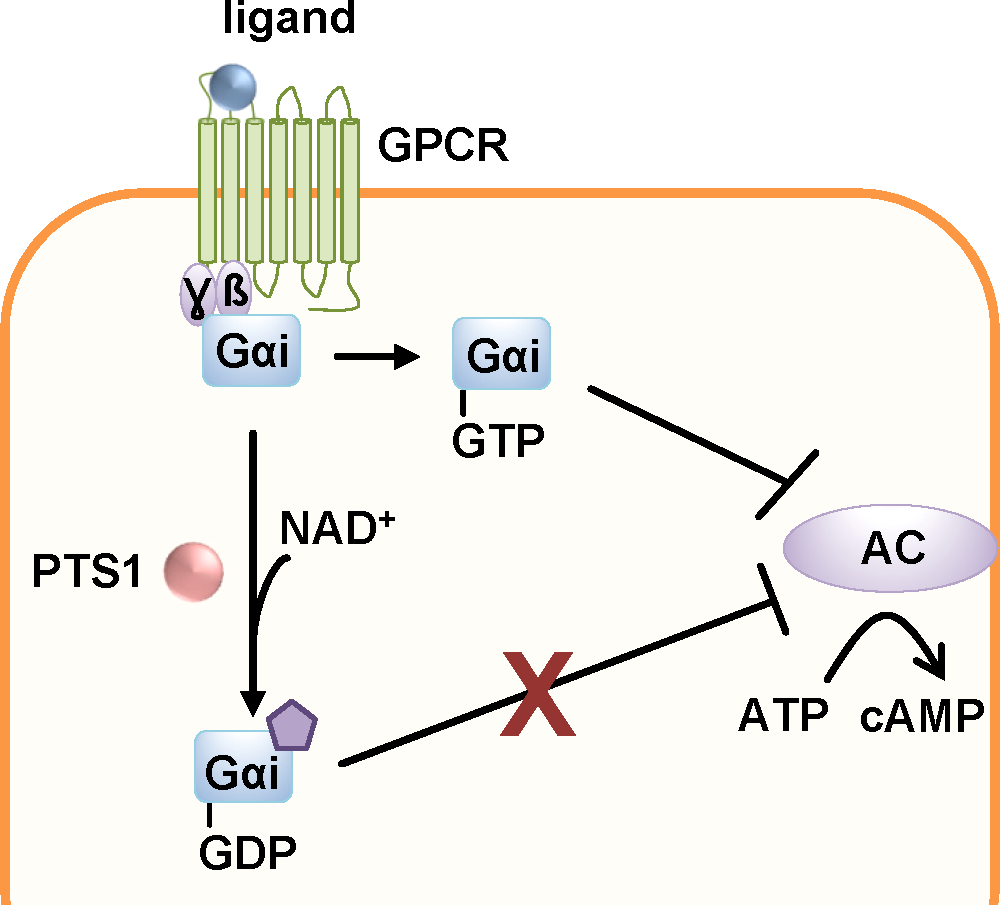
The enzyme subunit of pertussis toxin, PTS1, covalently transfers an ADP-ribose moiety from the co-substrate NAD^+^ onto the α-subunits of inhibitory G proteins (Gαi) of G protein coupled receptors (GPCR). The thereby inactivated Gαi can no longer down-regulate the adenylate cyclase (AC) upon GPCR activation. This leads to disturbed cAMP signaling (Locht et al., 2011).

As an AB_5_ toxin, PT consists of an enzymatically active A-subunit, PTS1 and a non-covalently bound pentameric B-subunit (**Figure 1**). The B-pentamer consists of subunits S2-5 with two molecules of S4 (Tamura et al., 1982). Assembly of the AB_5_ holotoxin occurs in the periplasm of the bacteria. PT binds to sialic acid structures that are present on many glycolipids and glycoproteins as terminal carbohydrates (Witvliet et al., 1989). After receptor-mediated endocytosis, PT takes a retrograde route through the Golgi network to the ER (**Figure 2**) (Plaut and Carbonetti, 2008). In the ER, binding of ATP to the central pore of the B-pentamer leads to the release of PTS1 from the holotoxin (Burns and Manclark, 1986; Hazes et al., 1996). Released PTS1 is thermally unstable and unfolds thereby it is recognized by the ER associated degradation pathway (ERAD) (Pande et al., 2006; Banerjee et al., 2016). ERAD transports unfolded or misfolded proteins through translocon pores into the cytosol where they are degraded. In its unfolded, linear conformation, PTS1 is a substrate for ERAD and can be transported through the narrow membrane-spanning translocon pore. Proteins transported through ERAD usually undergo ubiquitinylation at lysine residues which marks them for proteasomal degradation. The lack of lysine residues protects PTS1 from subsequent ubiquitinylation and therefore proteasomal degradation (Worthington and Carbonetti, 2007). In the cytosol, PTS1 catalyzes the covalent transfer of an ADP-ribose moiety from its co-substrate NAD^+^ onto the α-subunits of inhibitory G proteins (Gαi) of G protein coupled receptors (GPCRs) (Katada and Ui, 1982; Bokoch et al., 1983). Gαi is a negative regulator of the adenylate cyclase. ADP-ribosylation inhibits Gαi and therefore leads to increased cAMP levels upon receptor stimulation resulting in disturbed cAMP signaling (**Figure 6**).

**Figure 7:**
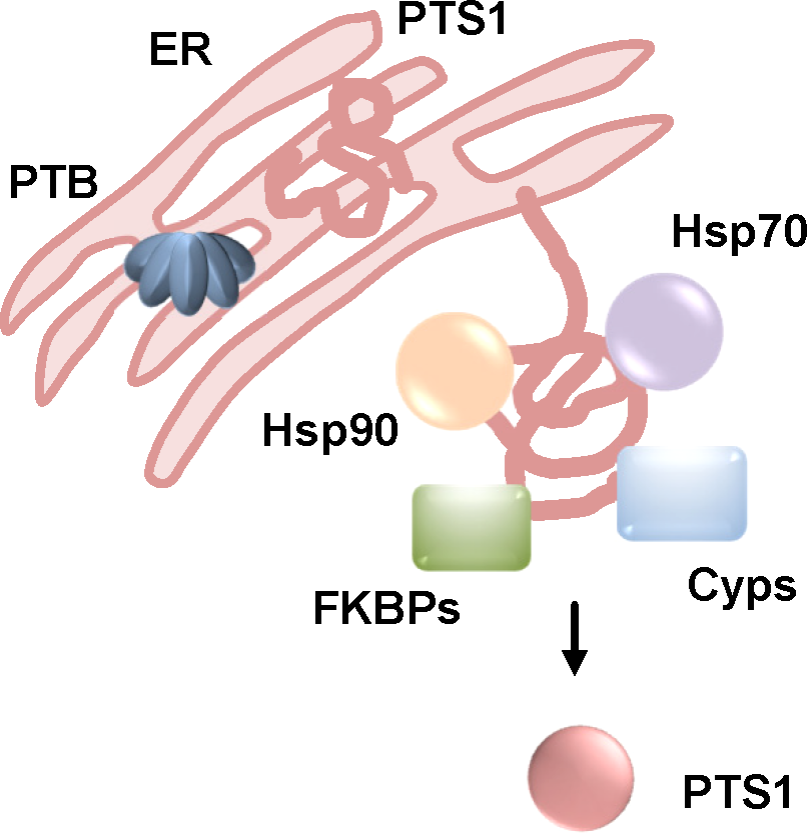
The activity of Hsp90, Hsp70, Cyps and FKBPs is required to the uptake of PTS1 into cells. After receptor-mediated endocytosis, PT is transported through the Golgi to the ER where PTS1 is released from the B-oligomer (PTB). This leads to unfolding of PTS1 due to thermal instability. Unfolded PTS1 is transported into the cytosol by the ER-associated degradation pathway. Upon inhibition of Hsp90, Hsp70, Cyps and FKBPs, intoxication of cells by PT is reduced, less PTS1 is detected in cells. For inhibition of Hsp90 and Cyps it was shown that less PTS1 molecules reach the cytosol suggesting that assistance of PTS1-translocation from the ER to the cytosol by chaperones and PPIases is the common underlying mechanism (Ernst et al., 2018a; Kellner et al., 2019; Ernst et al., 2021a; Kellner et al., 2021). (FKBPs, FK506 binding proteins; Cyps, cyclophilins).

The uptake of PT also depends on the activity of host cell factors Hsp90, Hsp70, Cyps and FKBPs (**Figure 7**) (Ernst et al., 2018a; Kellner et al., 2019; Ernst et al., 2021a; Kellner et al., 2021). Inhibition of these chaperones/PPIases in cells resulted in a reduced amount of ADP-ribosylated Gαi but had no effect on enzyme activity *in vitro* i.e. ADP-ribosylation of recombinant Gαi by PTS1. Binding of PT to cells was not affected by the inhibitors as well. To investigate if the inhibitors also prevented PT-mediated effects on cAMP signaling, a novel bioassay, the interference in Gαi-mediated signal transduction (iGIST) assay, was used (Paramonov et al., 2020; Ernst et al., 2021a). This assay is based on HEK293 cells that expresses the somatostatin receptor 2 (SSTR2), which is a Gαi-coupled GPCR sensitive to PT, together with a cAMP sensitive luciferase. Activation of SSTR2 by the high affinity agonist octreotide counteracts a simultaneous activation of the adenylate cyclase by its known activator forskolin. This results in down-modulated cAMP signaling that is detected by the intracellular cAMP sensitive luciferase. Treatment of cells with PT leads to inactivation of Gαi and in this case application of forskolin and octreotide result in increased cAMP levels because the down-modulation induced by SSTR2-activation is no longer possible (Paramonov et al., 2020). Rad and VER effects could not be investigated because they caused decreased cAMP levels on their own in this assay. However, CsA and FK506 prevented the PT-mediated cAMP increase in this kinetic bioassay (Ernst et al., 2021a).

In fluorescence microscopy experiments, chaperone/PPIase inhibitors caused a reduced signal of PTS1 in cells compared to cells treated with only PT suggesting that they interfere with the uptake of PTS1 into the cytosol (Ernst et al., 2018a; Ernst et al., 2021a). In another study, digitonin-based cell fractionation of PT-treated cells was performed in the presence or absence of the Hsp90 inhibitor geldanamycin (Kellner et al., 2019). This assay allows the separation of cells in cytosol and membrane fractions. PTS1 was detected in the cytosolic fractions by surface plasmon resonance using an anti-PTS1 antibody. The results showed that in the presence of geldanamycin less PTS1 is detected in the cytosol of PT-treated cells. To confirm that Hsp90 facilitates the translocation of PTS1 from the ER into the cytosol but does not interfere with trafficking of PT to the ER, CHO cells were transfected with a plasmid for expression of PTS1 directly in the ER. Thereby other steps of intracellular uptake are bypassed and the translocation of PTS1 into the cytosol can be analyzed in an isolated manner. Here too, geldanamycin as well as CsA led to a reduced amount of PTS1 in the cytosol of digitonin-fractionated cells (Kellner et al., 2019; Kellner et al., 2021).

A direct interaction of PTS1 with Hsp90, Hsc/p70, the Cyp isoforms CypA and Cyp40 as well as the FKBP isoforms FKBP51 and 52 but not FKBP12 were detected by dot blot assay (Ernst et al., 2018a; Ernst et al., 2021a). Moreover, PTS1 showed a reduced interaction with FKBP51/52 fragments only containing the PPIase domain. This suggests that PTS1 not only interacts with the PPIase domain but that other domains of the large FKBP isoforms might be required. The proximity ligation assay also revealed a close proximity of PTS1 with Cyp40, FKBP51, Hsp70 and Hsp90 in cells. A strong and robust signal in this assay was detected after 30 mins of pulse-chase incubation of cells with PT which was still detectable after 24 or 48 h indicating that the activity of chaperones/PPIases might not only be required for translocation from the ER to the cytosol but for continued stabilization of PTS1 in cytosol (Ernst et al., 2021a).

An inhibitory effect of CsA on the uptake of PTS1 into cells of a primary human bronchial airway epithelium model was shown (Ernst et al., 2021a). This airway epithelium is generated from human primary basal cells differentiated into different cell types such as ciliated and secretory cells that are grown at air-liquid interface conditions. Application of PT to the airway epithelium resulted in detection of PTS1 selectively in secretory (CC10 or MUC5B positive) cells but not in ciliated (ß-IV-tubulin positive) cells. Treatment of the airway epithelium with CsA but not Rad or FK506 caused a reduced signal for PTS1 in fluorescence microscopy experiments suggesting that uptake of PTS1 into the cytosol is also inhibited by CsA in this human airway epithelium model (Ernst et al., 2021a).

Taken together, translocation of PTS1 into the cytosol of target cells depends on the activity of Hsp90, Hsp70, Cyps and FKBPs. Inhibition of these host cell factors protected cells from PT-intoxication and resulted in reduced amounts of PTS1 in the cytosol. The protective effect of Cyp-inhibition was also shown in a primary human bronchial airway epithelium model.

### Cholera toxin

CT is one of the main virulence factors produced by *V. cholerae* (Haan and Hirst, 2004; Sánchez and Holmgren, 2008) and the causative agent for severe watery diarrhea that is characteristic for cholera (Haan and Hirst, 2004; Sack et al., 2004). The massive loss of water can be life-threatening. Without treatment, death can occur within hours after the first symptoms (Harris et al., 2012). According to the WHO position paper in 2017, an estimated 2.86 million cases of cholera occur annually in endemic countries resulting in an estimated 95 000 deaths (WHO position paper, 2017). Treatment against cholera consists of replacing lost fluids and electrolytes and in severe cases antibiotic treatment against the toxin-producing bacteria.

**Figure 8:**
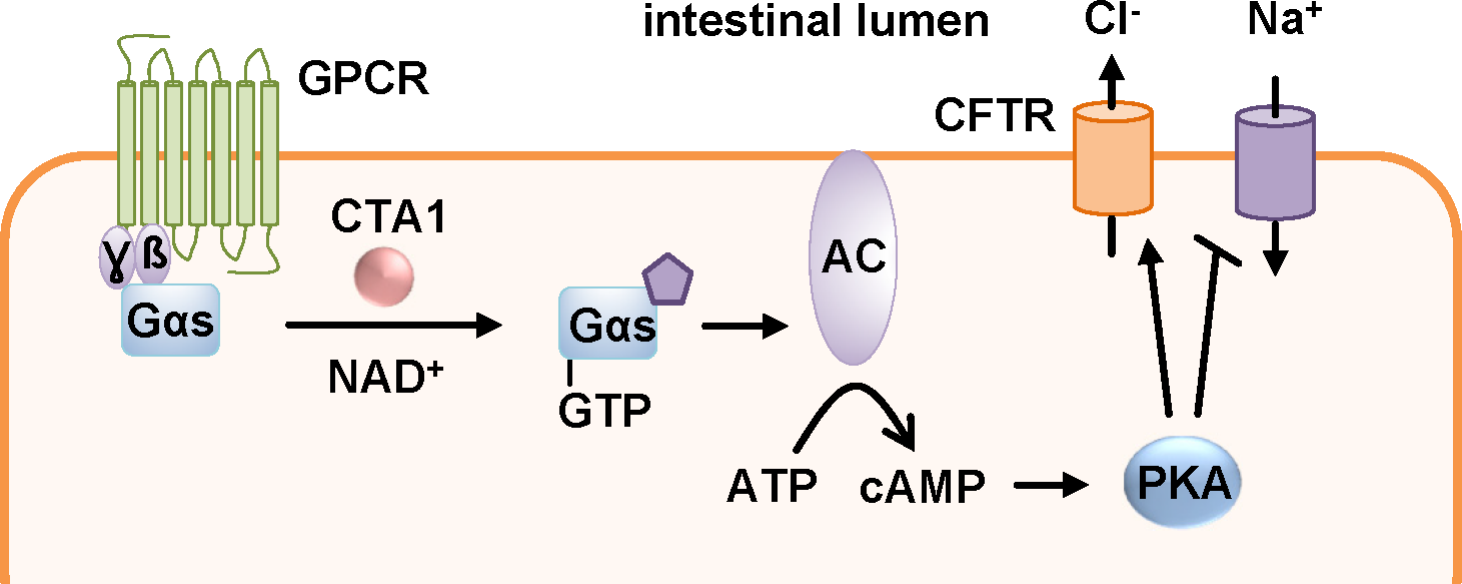
The enzyme subunit of cholera toxin, CTA1, ADP-ribosylates the α-subunits of stimulatory G proteins (Gαs) of G protein coupled receptors (GPCR). This leads to a constitutively active adenylate cyclase (AC) and a massive increase in intracellular cAMP. Activation of protein kinase A (PKA) results in inactivation of sodium channels and stimulation of the cystic fibrosis transmembrane regulator (CFTR) leading to secretion of chloride ions into the intestinal lumen. This results in extensive water secretion causing the watery diarrhea characteristic of cholera (Haan and Hirst, 2004).

*V. cholerae* is spread *via* a direct fecal-oral contamination or by ingestion of drinking water with fecal contaminations and then colonizes the small intestine. Here, CT is secreted into the intestinal lumen. As an AB_5_-toxin, CT consists of five B-subunits that form ring-shaped oligomers (B-oligomer, CTB) and an A-subunit that comprises the catalytic activity, CTA1, as well as a helical linker CTA2 (**Figure 1**). CTA1 and CTA2 are expressed as one polypeptide chain that is nicked post-translationally but still linked by a disulfide bond. CTA2 interacts non-covalently with the B-oligomer in the periplasm of the bacteria thereby forming the AB_5_-holotoxin. CTB facilitates the binding to the cellular receptor, GM1 gangliosides (Zhang et al., 1995; Haan and Hirst, 2004; Sánchez and Holmgren, 2008). Like PT, CT travels retrogradely to the ER (**Figure 2**) (Wernick et al., 2010). Reduction of the disulfide bond by protein disulfide isomerase in the ER leads to the release of CTA1 from the holotoxin (Majoul et al., 1997; Orlandi, 1997; Tsai et al., 2001; Taylor et al., 2011b; Taylor et al., 2014). Released CTA1 spontaneously unfolds under physiological temperature due to thermal instability (Pande et al., 2007; Taylor et al., 2011a). Transport to the cytosol occurs through the ERAD pathway and like PT, CT contains no lysine residues thereby being protected from ubiquitin-dependent degradation (Rodighiero et al., 2002; Teter and Holmes, 2002; Teter et al., 2003; Massey et al., 2009; Banerjee et al., 2010; Taylor et al., 2011a). In the cytosol, CTA1 ADP-ribosylates the α-subunits of stimulatory G proteins (Gαs) of GPCRs thereby activating Gαs (**Figure 8**). This leads to a stimulation of the adenylate cyclase and therefore to a massive increase if intracellular cAMP which in turn activates protein kinase A. Subsequently, protein kinase A leads to opening of the chloride channel, cystic fibrosis transmembrane regulator (CFTR). In enterocytes of the intestine, this leads to chloride release into the intestinal lumen which is followed by extensive water secretion and therefore causes the watery diarrhea (Haan and Hirst, 2004; Sánchez and Holmgren, 2008).

Also, for CT, a requirement of Hsp90 for translocation of its enzyme subunit CTA1 from the ER to the cytosol was demonstrated. The direct interaction of Hsp90 with CTA1 was ATP-dependent and inhibited by the Hsp90-inhibitor geldanamycin (Taylor et al., 2010). Interaction was analyzed at 37 °C degree suggesting that Hsp90 interacts with the unfolded CTA1. Moreover, in cells treated with geldanamycin the CT-mediated increase in cAMP was inhibited. If CTA1 was expressed directly in the cytosol of cells, Hsp90 inhibition had no effect on the cytopathic activity (Taylor et al., 2010). Knock-down of Hsp90 strongly protected cells from CT-intoxication demonstrating the crucial role of Hsp90 for the intoxication process. Digitonin-based cell fractionation showed that in the presence of geldanamycin less CTA1 reaches the cytosol of cells indicating that Hsp90 is involved in facilitating the translocation of CTA1 from the ER to the cytosol (Taylor et al., 2010). Direct expression in the ER and subsequent translocation of CTA1 into the cytosol revealed that upon Hsp90 inhibition again less CTA1 molecules reached the cytosol. This further confirms that Hsp90 is required for the translocation step rather than for any upstream steps of toxin uptake (Taylor et al., 2010). Geldanamycin also inhibited CT-mediated effects in an ileal loop model of intoxication. Therefore, CT was injected into surgically sealed sections of rat intestines. CT causes a distended morphology due to water accumulation. In the presence of geldanamycin, this CT-induced distended morphology was significantly reduced compared to intestinal loops treated only with CT indicating protection from intoxication in this pathophysiologically relevant ileal loop model (Taylor et al., 2010).

The role of Hsp90 for CT-intoxication was further elucidated and a co- as well as post-translocation role of Hsp90 was shown (Burress et al., 2014). Hsp90 not only facilitates the translocation of CTA1 into the cytosol but also refolds disordered CTA1 into its active conformation. This was shown by isotope-edited Fourier transform infrared spectroscopy (FTIR). An ATP-derivative that cannot be hydrolyzed inhibited refolding of CTA1 by Hsp90 as well as translocation of CTA1 from partially purified CT-loaded ER. Binding of the ATP-derivative/Hsp90 complex to CTA1 was not affected. This indicates that ATP hydrolysis by Hsp90 is required for CTA1 refolding and its translocation (Burress et al., 2014). Moreover, it was shown that incubation of disordered CTA1 at 37°C with Hsp90 and ATP results in a gain of function conformation. This was shown by significantly enhanced *in vitro* enzyme activity of CTA1 at 37°C in the presence of Hsp90 and ATP (Burress et al., 2014). Further *in vitro* interaction analysis by surface plasmon resonance revealed that Hsp90 is not released after CTA1 folding but continues to bind to CTA1 even in the presence of Hsp90 co-factors such as Hsp40 or p23 or other host factors represented by addition of cytosolic extracts to the interaction partners (Burress et al., 2014). Based on these data, an Hsp90-dependent ratchet mechanism was proposed combining extraction and translocation of CTA1 from the ER to the cytosol with its refolding. Thereby, a unidirectional transport will be favored by preventing the folded parts of CTA1 to translocate back into the ER (Taylor et al., 2010; Burress et al., 2014).

By generating overlapping peptides of CTA1, two binding sites for Hsp90 with the amino acid sequences RPPDEI and LDIAPA were identified (Kellner et al., 2019). RPPDEI comprises residues 11-16 and LDIAPA 153-158 of CTA1. Expression of CTA1 mutants, lacking either one of the identified motifs, in the ER of cells showed that both mutants were not detected in the cytosol after digitonin-based cell fractionation suggesting that both binding sites are required for toxin translocation into the cytosol. Moreover, CTA1 constructs with point mutations in the binding motif revealed the RPP as key residues for efficient translocation of CTA1 from the ER to the cytosol (Kellner et al., 2019). Interestingly, the RPPDEI motif was also found in the sequence of other toxins translocating from the ER such as PT but not in toxins translocating from endosomes such as DT or clostridial binary toxins. This suggests that recognition of endosome-translocating toxins by Hsp90 appears to be different from how ER-translocating toxins interact with Hsp90 (Kellner et al., 2019). In contrast to PT, DT and clostridial binary toxins, it was shown that inhibition of Cyps by CsA did not protect cells from CT-intoxication (Burress et al., 2019).

It was further hypothesized that the proline isomerization in the RPPDEI motif is crucial for interaction with Hsp90 and that Hsp90 only recognizes cis prolines in this motif (Kellner et al., 2021). Folded CTA1 contains trans prolines. Unfolding might lead to cis prolines since this is the preferred conformation of proline-proline bonds in proteins. Refolding by Hsp90 could lead to maintaining of the cis conformation which might also explain the continued interaction of Hsp90 with CTA1 even after translocation and refolding (Burress et al., 2014; Kellner et al., 2021). The authors moreover proposed that endosome-translocating toxins lacking the RPPDEI motif might require PPIases for trans-to-cis isomerization to prepare them for interaction with Hsp90 (Kellner et al., 2021).

**Figure 9:**
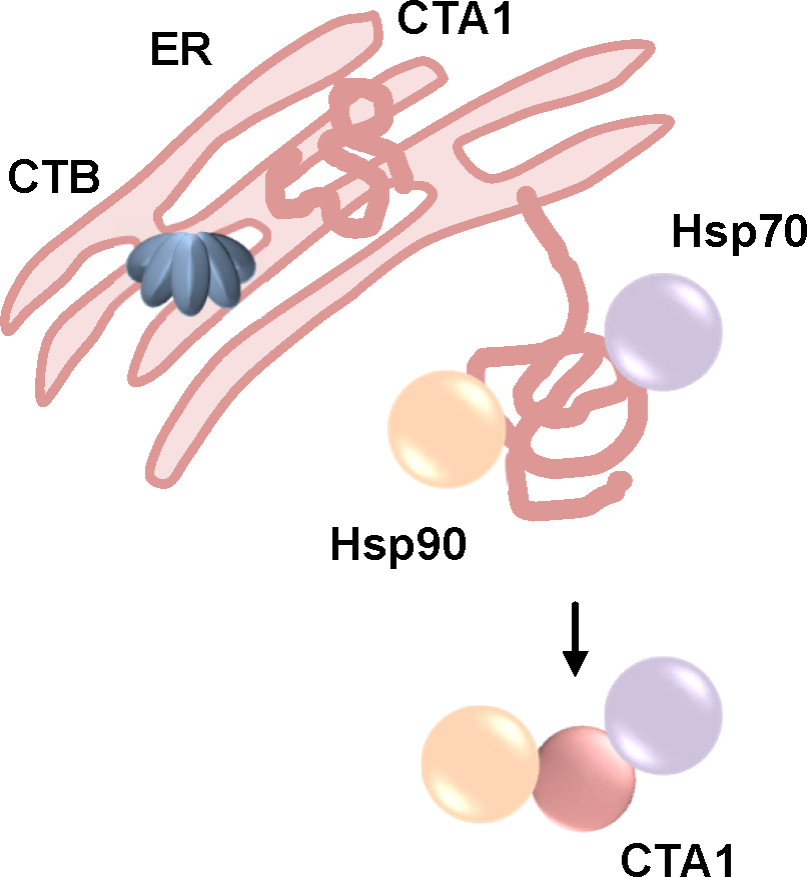
The activity of Hsp90, Hsp70 but not Cyps is required to the uptake of CTA1 into cells. After receptor-mediated endocytosis, CT is transported through the Golgi to the ER where CTA1 is released from the B-oligomer (CTB). This leads to unfolding of CTA1 due to thermal instability. Unfolded CTA1 is transported into the cytosol by the ER-associated degradation pathway. Upon inhibition of Hsp90 or Hsp70 intoxication of cells by CT is reduced and less CTA1 is detected in the cytosol (Taylor et al., 2010; Burress et al., 2014; Burress et al., 2019; Kellner et al., 2019; Kellner et al., 2021).

In addition to Hsp90, Hsc70 is also involved in translocation and refolding of CTA1 (**Figure 9**) (Burress et al., 2019). Inhibition of both Hsc70 and Hsp70 by the inhibitor PES significantly reduced the CT-mediated cAMP increase compared to cells treated with CT only. Interestingly, knock-down of either Hsc70, Hsp70 or the Hsp90 adaptor protein Hop did not result in inhibition of CT-mediated cAMP increase suggesting that Hop is not involved in CT uptake and that the known redundant functions of Hsc and Hsp70 probably compensate the others knock-down. Moreover, PES-treatment resulted in reduced translocation of CTA1 that was expressed directly in cells and redirected to the ER (Burress et al., 2019). A direct binding of Hsc70 to both, folded and disordered, CTA1 was demonstrated. This interaction was independent of Hsp40, a protein often required for delivery of client proteins to Hspc70. Binding of Hsp90 and Hsc70 occurs at different sites of CTA1 because Hsp90 was able to bind to the pre-formed Hsc70/CTA1 complex and vice versa. By generating overlapping peptides of CTA1, it was revealed that Hsc70 binds to a specific sequence, YYIYVI, in CTA1 (Burress et al., 2019). It was also shown by structure analysis that Hsc70 facilitates refolding of CTA1 (Burress et al., 2019).

Taken together, it was shown that Hsp90 and Hsp70 but not Cyps are required for translocation of CTA1 into the cytosol of target cells. Hsp90 and Hsp70 not only facilitate the translocation but also the refolding of CTA1 into its active conformation. Moreover, Hsp90 not only interacts during but also after translocation most likely to stabilize the active conformation of the thermally instable CTA1.

### Effect of novel derivatives of PPIase inhibitors on bacterial toxins

The use of established inhibitors was essential to investigate and unravel the role of chaperones and PPIases during uptake of bacterial toxins. However, these inhibitors have some drawbacks regarding potential therapeutic application. CsA and FK506 are potent immunosuppressive drugs that are used for example after organ transplantation to avoid organ rejection. In context of an infection with bacteria, downregulation of the immune system is not be desired. For CsA, non-immunosuppressive derivatives such as VK112 (Prell et al., 2013; Braun et al., 2022) or NIM811 (Rosenwirth et al., 1994) were generated that lack the typical immunosuppressive effect while maintaining inhibition of the PPIase activity of Cyps. VK112 protected cells from intoxication with the clostridial binary toxins C2, iota and CDT toxin (Ernst et al., 2015), as well as with DT (Schuster et al., 2017) and PT (Ernst et al., 2018a). Inhibition of pH-dependent membrane translocation by VK112 was exemplarily shown for the C2 toxin (Ernst et al., 2015). A cell-impermeable CsA derivative, MM284 (Malesevic et al., 2013), had no inhibitory effect on intoxication with C2 toxin or DT indicating that extracellular Cyps do not play a role for their mode of action (Ernst et al., 2015).

Moreover, the effect of NIM811 was tested in a *B. pertussis* infection model using infant mice (Ernst et al., 2021a). In contrast to investigating adult mice, infant mice more accurately recapitulate hallmarks of severe disease observed in humans (Scanlon et al., 2017). One hallmark in human disease is severe leukocytosis which is associated with expression of PT as well as with fatal outcome in newborns and infants (Mattoo and Cherry, 2005; Scanlon et al., 2019). Therefore, 7-day old mice were infected with a wild type *B. pertussis* strain producing PT *via* aerosol. This was followed by intranasal treatment with CsA, NIM811 or vehicle. CsA or NIM811 had no effect on the bacterial burden that was analyzed from homogenized lung tissue 7 days post-infection. However, CsA and NIM811 both significantly inhibited leukocytosis which was measured by determining the white blood cell count from harvested blood by using a hemocytometer. VK112 or NIM811 as specifically designed derivatives represent interesting starting points for the development of novel therapeutic strategies to treat diseases that are caused by bacterial toxins that depend on the activity of Cyps for their cellular uptake and therefore for their cytotoxic effect. A major advantage of such derivatives is that CsA is an already licensed drug with a known pharmacokinetics and safety profiles.

To determine which isoforms of Cyps or FKBPs play a crucial role during toxin uptake, isoform-specific inhibitors are of use. For FKBP51, an isoform-specific inhibitor called SAFit1 was used to investigate the uptake of DT (Gaali et al., 2015; Schuster et al., 2017; Kolos et al., 2018; Hähle et al., 2019). Cells were pre-incubated with SAFit1 and then challenged with DT. DT intoxication was monitored by specific morphological changes induced by DT. In the presence of FK506 or SAFit1, less DT-intoxicated cells were observed compared to cells treated only with DT. This result indicated that FKBP51 is functionally involved in the cellular uptake of DT (Schuster et al., 2017). Using isoform-specific inhibitors contributes to further elucidation of the precise mechanism of toxin translocation.

## Common characteristics of chaperone/PPIase-dependent toxins and conclusion

A functional role of Cyps, FKBPs, Hsp90 and Hsc/p70 has been demonstrated for several bacterial AB-type toxins. Common characteristics as well as differences in the requirement and interaction of the various toxins with the protein folding helper enzymes have been observed. During the course of investigation, the hypothesis that the enzyme activity of a toxin determines if chaperones and PPIases are required for cellular uptake. **Table 1** gives an overview of toxins for which the requirement of chaperones and PPIases was investigated. Most toxins that need chaperones and PPIases for their uptake are ADP-ribosyltransferases including clostridial binary toxins, DT and PT. Toxins such as *C. difficile* TcdA and TcdB or the *Bacillus anthracis* lethal toxin which are not ADP-ribosyltransferases are independent of Hsp90, Cyps and FKBPs for their cellular uptake (Haug et al., 2003a; Kaiser et al., 2009; Zornetta et al., 2010; Dmochewitz et al., 2011; Kaiser et al., 2012; Steinemann et al., 2018). Investigation of fusion toxins as well as artificial transport of isolated enzyme subunits further supported this hypothesis. The fusion toxin LF_N_DTA transported *via* PA, the B-component of the anthrax toxin, into cells requires Hsp90 and Cyps to reach the cytosol. The wildtype lethal toxin that has metalloprotease activity and requires the same B-component, PA, for its uptake is independent of Hsp90/Cyps (Zornetta et al., 2010; Dmochewitz et al., 2011).

**Table 1:** Overview of requirement of chaperones/PPIases for uptake of bacterial AB-type toxins.

Enzyme activity	Toxin	Hsp90	Hsp/Hsc70	Cyps	FKBPs	References
ADP-RT	C2 toxin	✓	✓	✓	✓	(Haug et al., 2003a; Kaiser et al., 2009; Kaiser et al., 2012; Ernst et al., 2015; Ernst et al., 2017a)
	Iota toxin	✓	✓	✓	✓	(Haug et al., 2004; Kaiser et al., 2011; Kaiser et al., 2012; Ernst et al., 2015; Ernst et al., 2016; Ernst et al., 2017a)
	CDT toxin	✓	✓	✓	✓	(Kaiser et al., 2011; Kaiser et al., 2012; Ernst et al., 2015; Ernst et al., 2017a)
	PTC3 toxin	✓	n.a.	✓	✓	(Lang et al., 2014)
	DT	✓	✓	✓	✓	(Ratts et al., 2003; Dmochewitz et al., 2011; Schuster et al., 2017)
	PT	✓	✓	✓	✓	(Ernst et al., 2018a; Kellner et al., 2019; Ernst et al., 2021a; Kellner et al., 2021)
	CT	✓	✓	–	n.a.	(Taylor et al., 2010; Burress et al., 2014; Burress et al., 2019; Kellner et al., 2019; Kellner et al., 2021)
	C2IN-C3lim + C2IIa	✓	n.a.	✓	✓	(Pust et al., 2007; Kaiser et al., 2009; Kaiser et al., 2012)
	LF_N_-DTA + PA	✓	n.a.	✓	n.a.	(Dmochewitz et al., 2011)
	HisTccC3hvr + PA	✓	✓	✓	✓	(Lang et al., 2014; Ernst et al., 2017a)
MP	BoNT, TeNT	✓	–	–	n.a.	(Azarnia Tehran et al., 2016; Pirazzini et al., 2018)
	AIP56	✓	n.a.	✓	–	(Rodrigues et al., 2019)
	LT	–	n.a.	–	–	(Haug et al., 2003b; Zornetta et al., 2010; Dmochewitz et al., 2011)
GT	TcdA, TcdB	–	n.a.	–	–	(Haug et al., 2003b; Kaiser et al., 2009; Dmochewitz et al., 2011; Kaiser et al., 2012; Steinemann et al., 2018)

(✓ = uptake of toxin depends on respective factor, n.a. = not analyzed). ADP-RT, ADP-ribosyltransferase; GT, Glycosyltransferase; MP, Metalloprotease.

Another ADP-ribosylating toxin, PTC3 produced by *Photorhabdus luminescens*, also requires the activity of Hsp90, Cyps and FKBPs for the translocation of its enzyme subunit into the cytosol (Lang et al., 2014). PTC3 shows a tripartite structure with a linker protein in addition to the A- and B-subunit. Moreover, PTC3 employs an elaborate syringe-like translocation machinery for injecting its enzyme subunit TccC3 into the host cell cytosol (Lang et al., 2010; Gatsogiannis et al., 2013). Moreover, the isolated enzyme domain of TccC3 is efficiently transported into the cytosol of target cells *via* PA. PA is used as a delivery system not only by fusing cargo proteins to the N-terminal part of its natural enzyme component lethal factor but also by addition of a His-tag to the cargo. This enables the interaction with PA and thereby transport and translocation of the His-tag protein into the cytosol (Blanke et al., 1996; Lang et al., 2010; Beitzinger et al., 2012). Comparable to the uptake of LF_N_DTA *via* PA, translocation of the His-tagged enzyme domain of PTC3 *via* PA was also inhibited by Rad, CsA and FK506 although uptake of the wild type enzyme component lethal factor *via* PA was not affected by these inhibitors (Lang et al., 2014).

Another fusion protein, C2IN-C3lim, consists of the N-terminal part of the enzyme component C2I of C2 toxin fused to the C3 toxin of *C. limosum* (C3lim). C3lim is an ADP-ribosylating toxin but is only taken up efficiently into monocyte derived cells (Barth et al., 2015). C2IN has no enzyme activity but facilitates the interaction with the C2IIa heptamer enabling the uptake of C2IN-C3lim into a variety of cell types. It was shown that uptake of C2IN *via* C2IIa is independent of Hsp90 and PPIases. However, if C2IN-C3lim requires the activity of Hsp90 and PPIases for efficient membrane translocation *via* C2IIa from endosomes to the cytosol (Pust et al., 2007; Kaiser et al., 2009, Kaiser et al., 2012). This further supports the hypothesis of a common Hsp90/PPIase-dependent membrane translocation for ADP-ribosylating toxins.

A role of Hsp90 for cellular uptake has been reported for toxins with different enzyme activities. Botulinum and tetanus neurotoxins are single chain, short-trip toxins with a metalloprotease activity. Both toxins require Hsp90 activity but are independent of Cyps and Hsc/Hsp70 suggesting that Hsp90 is more versatily involved in interactions with different bacterial AB-type toxins compared to requirement of PPIases (Azarnia Tehran et al., 2016; Pirazzini et al., 2018). A role of FKBPs was not investigated for these toxins. Moreover, the single-chain AB-toxin, AIP56, which is also a metalloprotease, depends on the activity of Hsp90 and Cyps but not FKBPs (Rodrigues et al., 2019). It was also shown that the ADP-ribosylating CT requires Hsp90 and Hsc/p70 but not Cyps for translocation and folding of the enzyme subunit (Taylor et al., 2010; Burress et al., 2014; Burress et al., 2019; Kellner et al., 2019; Kellner et al., 2021). However, the related PT, which is like CT a long-trip toxin and an ADP-ribosyltransferase, depends on Hsp90, Hsc/p70, Cyps and FKBPs (Ernst et al., 2018a; Kellner et al., 2019; Ernst et al., 2021a; Kellner et al., 2021). These results challenge the hypothesis of a common chaperone/PPIase dependent mechanism specific for ADP-ribosylating toxins. The requirement of chaperones/PPIases is rather more comprehensive including not only ADP-ribosylating toxins but also toxins with other enzyme activities such as metalloproteases. Moreover, the composition of chaperones and PPIases that are required for efficient toxin uptake are more variable and versatile with Hsp90 being the common denominator so far. How widespread the use of chaperones and PPIases by bacterial AB-type toxins is and what exactly determines a toxin’s dependency on chaperones/PPIases has to be revealed in future research.

## Author contributions

The author confirms being the sole contributor of this work and has approved it for publication.

## Funding

This work was supported by the Deutsche Forschungsgemeinschaft (DFG, German Research Foundation)—grant/project number 316249678-SFB 1279 (A07, funding to KE) and Pulmosens GRK 2203. KE is a fellow of the Margarete von Wrangell Habilitation program supported by the European Social Fund and Ministry of Science, Research and Art Baden-Wurttemberg.

## Conflict of interest

The author declares that the research was conducted in the absence of any commercial or financial relationships that could be construed as a potential conflict of interest.

## Publisher’s note

All claims expressed in this article are solely those of the authors and do not necessarily represent those of their affiliated organizations, or those of the publisher, the editors and the reviewers. Any product that may be evaluated in this article, or claim that may be made by its manufacturer, is not guaranteed or endorsed by the publisher.
